# Supplementary material for: Coordinated changes in gene expression kinetics underlie both mouse and human erythroid maturation
Source: Genome Biol. 2021 Jul 5;22:197. doi: 10.1186/s13059-021-02414-y (PMC8258993; doi:10.1186/s13059-021-02414-y)
Supplement: Supplementary file 1 — Additional file 1: Supplementary Figures. Supplementary Figures and corresponding legends. [file 13059_2021_2414_MOESM1_ESM.pdf]

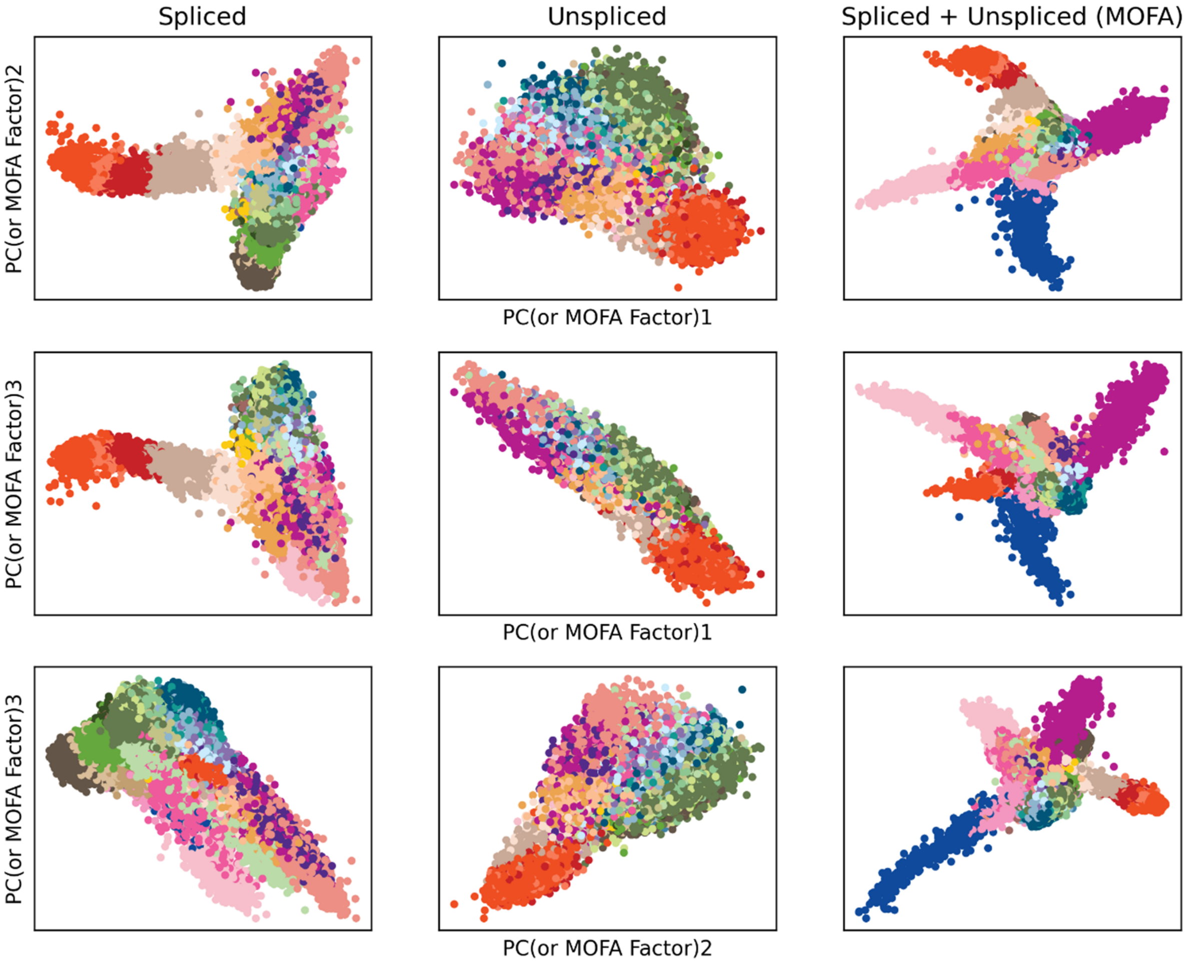

Fig. S1. Comparing variability-based dimensionality reduction methods. Dimensionality reduction with the first three principal components/MOFA factors using spliced reads alone (left), unspliced reads alone (middle) and both spliced and unspliced (right). Single-cell transcriptomes are colored by cell-type annotation; see Figure 1 for full legend.

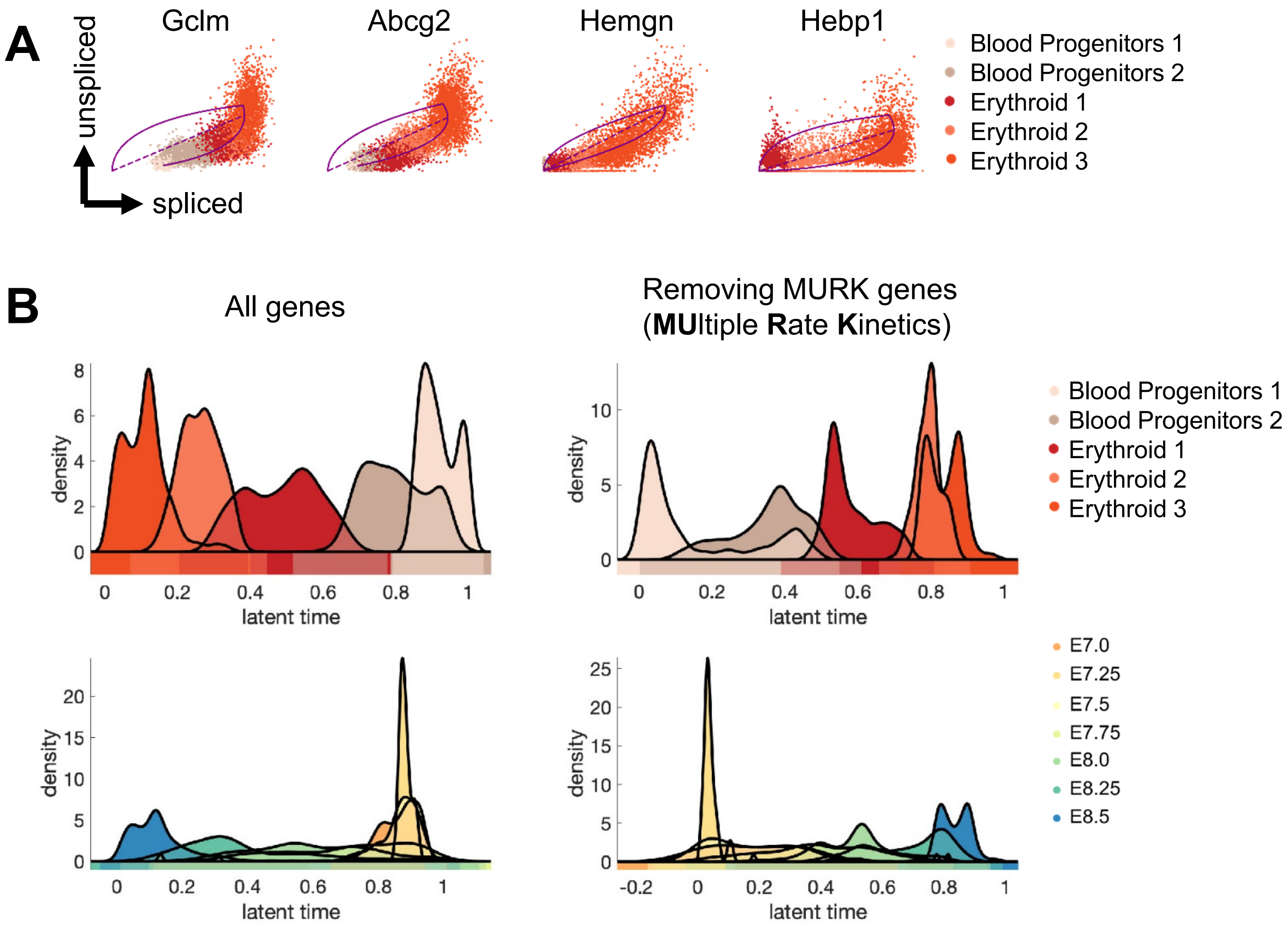

Fig. S2. Identification of MURK genes along yolk sac erythropoiesis.
A. Phase plots of representative scVelo driver genes, with scVelo model prediction overlaid (see also Additional File 2: Table S1).
B. Distribution of annotated cell type (top) and sampling time-point (bottom) along scVelo calculated latent time, using all genes (left panels) and after removing the MURK genes identified in Figure 3B-C.

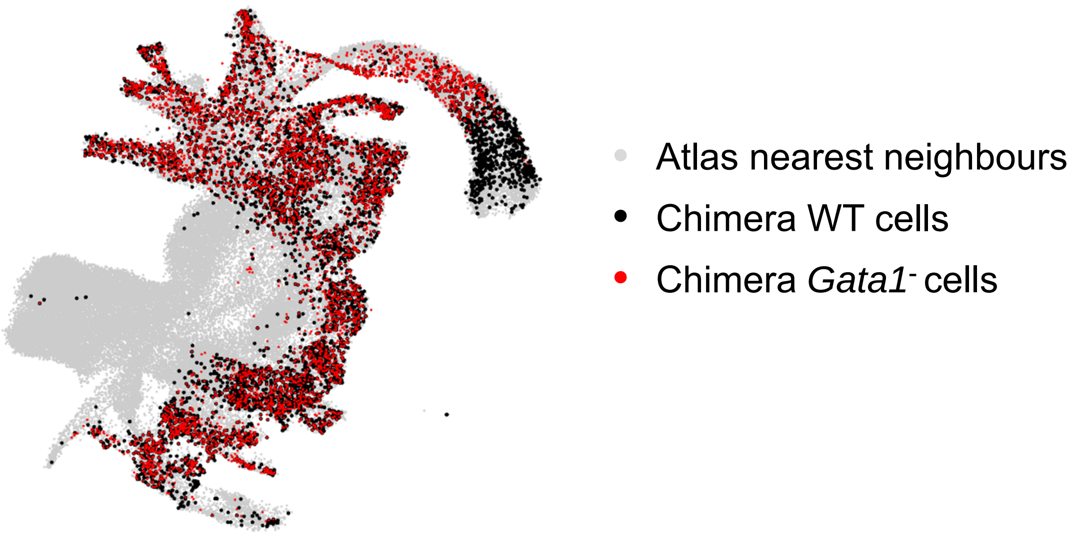

Fig. S3. Contribution to Chimera cells on the overall Atlas UMAP projection. Pijuan-Sala et al. (25) layout highlighting nearest neighbours of $Gata1^-$ chimeras. In red are nearest neighbours of $tdTom^+$ mutant cells, in black those of $tdTom^-$ wildtype cells. To compare with Figure 1A.

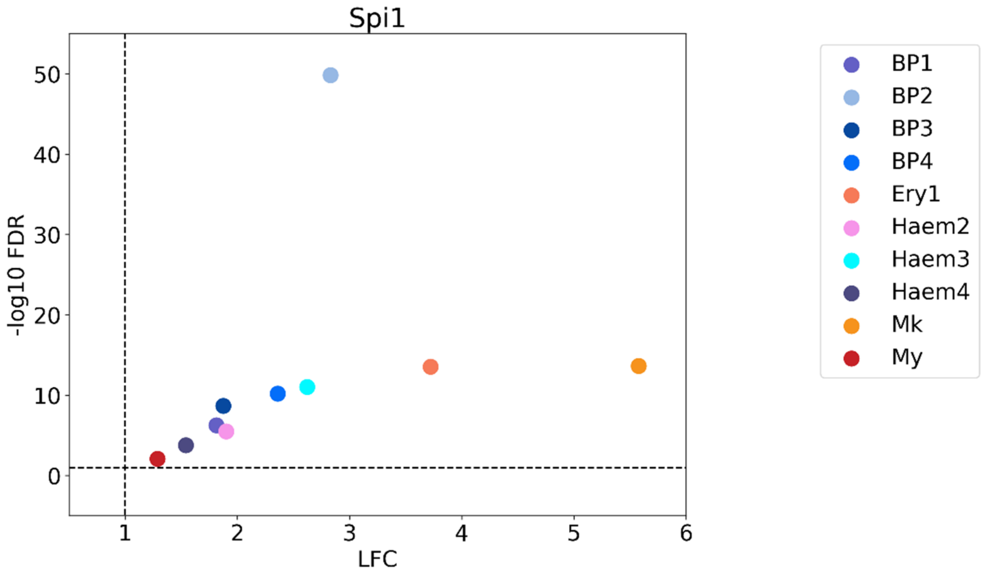

Fig. S4. *Spi1* is upregulated in Gata1⁻ yolk sac hematopoiesis.
Impact of *Gata1* knockout on *Spi1/PU.1* expression on the hematoendothelial cell types. X-axis: *Spi1* $\log_2$(fold-change) in Gata1⁻ vs WT chimera cells and Atlas nearest neighbours. Y-axis: $\log_{10}$(FDR).

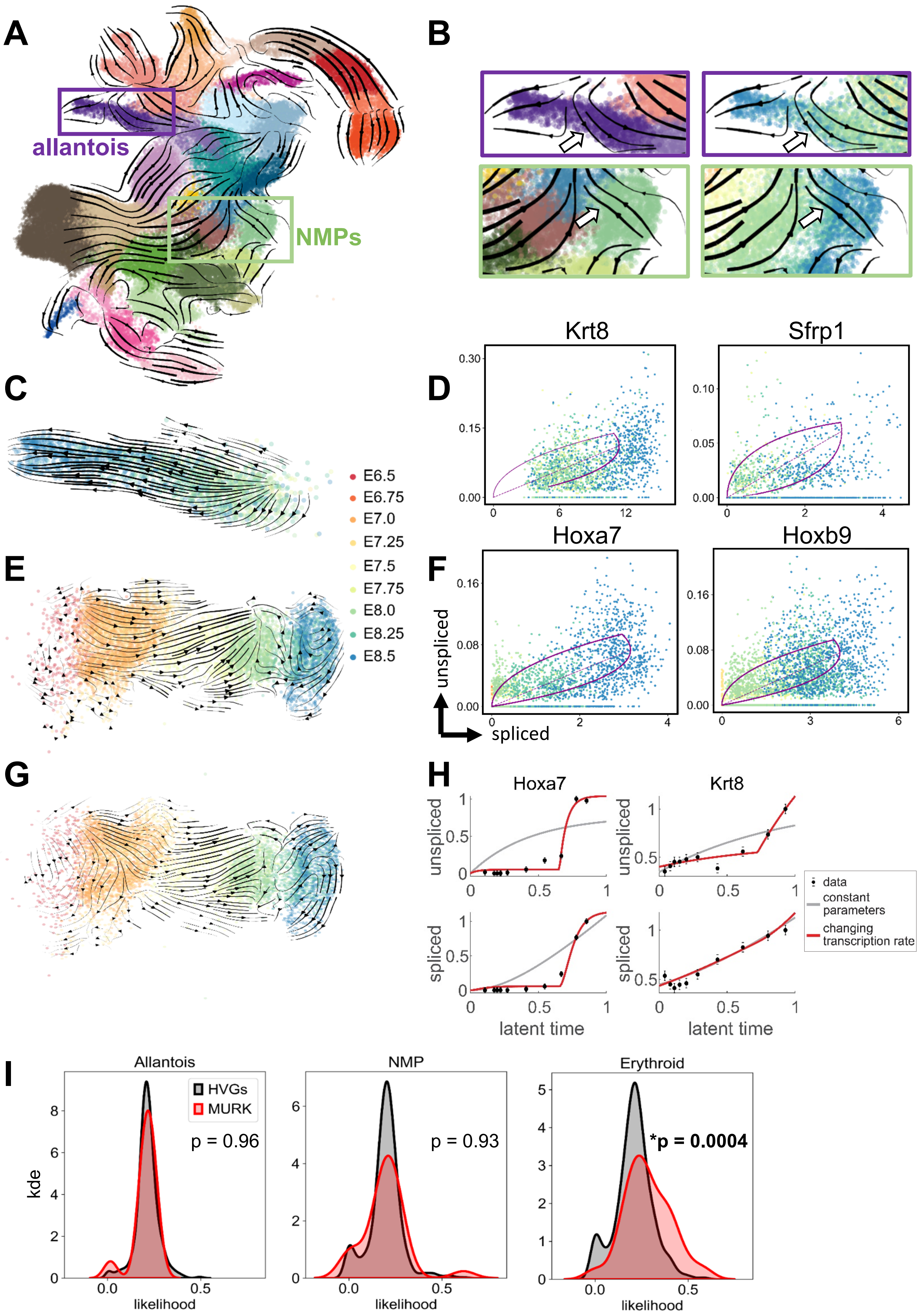
A
allantois
NMPs
B
C
E6.5
E6.75
E7.0
E7.25
E7.5
E7.75
E8.0
E8.25
E8.5
D
Krt8
Sfrp1
E
F
Hoxa7
Hoxb9
unspliced
spliced
G
H
Hoxa7
Krt8
unspliced
spliced
latent time
data
constant parameters
changing transcription rate
I
Allantois
NMP
Erythroid
HVGs
MURK
p = 0.96
p = 0.93
*p = 0.0004
kde
likelihood

Fig. S5. Identification of MURK genes in two additional developmental trajectories.

A. Pijuan-Sala et al. (25) layout with allantois and NMP cell types highlighted, where, as for the erythroid trajectory analysed in Figures 1-3, scVelo arrows run contrary to real-time sampling. See Figure 1 for full legend.

B. Close-up of regions highlighted in (A). Left: cells colored by cell-type; right: cells colored by collection time-point. White arrows: scVelo predictions running “backwards” in time.

C. UMAP representation of allantois cell subset. The overlaying arrows result from applying scVelo to this cell subset alone. In contrast to the predictions resulting from applying scVelo to the whole dataset (see A and B), implementing scVelo (including highly variable genes calculation) to this cell subset alone resulted in correct predictions, with scVelo arrows now in agreement with real-time sampling. This shows that scVelo is highly sensitive to the cell subset, and associated highly variable genes, used in the calculations.

D. Phase plots of two representative allantois MURK genes, with scVelo model prediction overlaid. Cells are colored by collection time-point.

E. UMAP representation of cells belonging to the NMP trajectory, previously calculated in (31), colored by collection time-point. The overlaying arrows result from applying scVelo to this cell subset, with “backwards” predictions at later time-points.

F. Phase plots of two representative NMP MURK genes, with scVelo model prediction overlaid.

G. Same UMAP as in E, overlaying arrows result from applying scVelo to this cell subset after NMP MURK gene exclusion, which improved the directionality of the arrows.

H. Fitting parameter change models to Krt8 (allantois MURK gene) and to Hoxa7 (NMP MURK gene). Grey line: model with constant parameters over latent time (scVelo assumption); red line: model with transcription rate change over latent time. This analysis suggests that in NMP and allantois, MURK genes may also be driven by transcription rate changes. See also Additional file 5: Supplementary Note.

I. Distribution of scVelo likelihood scores of highly variable genes for each trajectory subset (allantois, NMPs, erythroid), and MURK genes alone. The likelihood score reflects how well the spliced/unspliced counts for a given gene match with the underlying model assumptions. While the two curves are similar in the case of the allantois and NMP trajectories, a substantial proportion of the MURK genes identified in the erythroid trajectory had higher likelihood values than the remaining erythroid HVGs. p-values were calculated using two-sided Kolmogorov-Smirnov test, showing that the distribution of MURK genes is significantly different from the remaining HVGs in the case of the erythroid trajectory, in contrast to the other two trajectories considered, where likelihood distributions are similar. The high likelihood scores of erythroid MURK genes may explain their pronounced effect on arrow directionality upon scVelo implementation. kde: kernel density estimation.
