# Supplementary material for: Coordinated changes in gene expression kinetics underlie both mouse and human erythroid maturation
Source: Genome Biol. 2021 Jul 5;22:197. doi: 10.1186/s13059-021-02414-y (PMC8258993; doi:10.1186/s13059-021-02414-y)
Supplement: Supplementary file 7 — Additional file 7. Review history. [file 13059_2021_2414_MOESM7_ESM.docx]

Review History

**First round of review**

**Reviewer 1**

**Are you able to assess all statistics in the manuscript, including the appropriateness of statistical tests used?**

No, I do not feel adequately qualified to assess the statistics.

**Comments to author:**

In this manuscript, Barile et al. applied RNA velocity (an approach based on the ratio between unspliced and spliced RNA) to their previously established temporal scRNAseq data of mouse gastrulation and organogenesis with the goal of predicting the directionality of differentiation. Interestingly, they found that while scVelo was able to correctly predict some differentiation trajectories, it also failed on others, particularly erythropoiesis where it predicts a backward differentiation from later to earlier cell populations. Furthermore, the authors obtained the same result on another scRNAseq dataset from human fetal liver erythropoiesis. Then, they went on to demonstrate that this failure in predicting the correct direction of differentiation was due to specific genes (the MURK genes) that abruptly increase their transcription rate mid-way through erythroid differentiation, violating the assumption of a constant transcriptional rate that underlies sc Velo. Importantly, the authors identified those genes and showed that removing them is sufficient to correct the trajectory direction as inferred by scVelo. Finally, using their chimera-seq approach with Gata1 KO they demonstrate that Gata1 is involved in regulating some of the MURK genes. Furthermore, they found that the Gata1 KO leads to a block of erythroid differentiation and an increase in megakaryocytic differentiation. Finally, they detected an upregulation of Pu1 consistent with the Gata1/Pu1 antagonism.

Overall, this is an elegant and important study with several new findings. The finding of the importance of constant transcription rate for scVelo to correctly predict the direction of erythroid differentiation is very important for anyone planning to use scVelo. The identification of a coordinated change in transcriptional rate for specific genes in late erythroid differentiation is another important finding, permitted by the use of chimera-seq. I only have one question and one comment.

Point 1: Beside the "backward" erythroid trajectory, the authors have noticed other, time-related discrepancies in scVelo prediction, including mesoderm and endoderm cells. Are these other discrepancies also due to genes that display an abrupt change in transcription rate?

Conversely, I am wondering if the trajectories where scVelo is correct are completely devoid of genes that change their transcription rate? In other words: can velocity tolerate a certain number/proportion of genes changing their transcription rate and still be correct? These are important questions when deciding whether scVelo can be used in specific cases, and/or whether corrective measures could be used to improve scVelo algorithm.

Point 2: In the discussion, it is suggested that Gata1 is important for both cell fate choice (because of the increased in Pu1 expression and megakaryocyte differentiation) and cell fate stabilization (because of the gata1-dependent expression of some effector genes in late erythroid cells). While I agree that the data strongly support a cell fate decision role for Gata1, there is no evidence that the increased expression of effector genes (e.g. the globin genes) in late erythroid cells plays any role in cell fate stabilization. I would suggest to remove this from the Discussion.

Minor point: On Figures 1 and 2, the arrows from scVelo are very difficult to see. It would help to increase their size.

**Reviewer 2**

**Are you able to assess all statistics in the manuscript, including the appropriateness of statistical tests used?**

Yes, and I have assessed the statistics in my report.

**Comments to author:**

The paper by Barile et al presents an interesting and innovative interrogation of a large scRNA-seq data set of mammal erythropoiesis. The most innovative part of the study, in my opinion, is the use of RNA velocity in a "prospective" fashioon, i.e. to predict cell transitions and, crucially, to exploit predictive failures to gain new insights in the biology. The authors observe a systematic failure of scVelo to correctly predict some transitions, and then attempt to reconstruct the source of this discrepancy by indentifying genes whose expression behaviour appears to violate the modelling assumptions (constant expression kinetics) underlying the model. In doing so, they identify 89 Multiple Rate Kinetics (MURK) genes which exhibit a sharp change in kinetics. Removing these genes greatly improves the performance of scVelo, and also enables the authors to derive several interesting biological observations.

Overall, the paper is well written and interesting, and imo deserves publication. There are two major contributions: the identification of the cluster of MURK genes might be of biological value in itself (I am not an expert on this and I will leave it to others to comment on the more bio aspects of the paper). But to me the more interesting aspect is the use of model failure (scVelo) to learn something new. On this I have a few comments:

- the derivation of the "method" to identify the MURK genes seems mostly heuristic. Which is fine, but I would then want to understand how sensitive it is to arbitrary parameters (e.g. what if, instead of requiring the slope to be positive, one would require it to be greater than say 0.1? what if the p-value threshold is taken to be 0.01 instead of 0.05, or 0.1?).

- Ultimately, I imagine one could conceive a more principled way to correct deviations from model assumptions; throwing away the MURK genes in RNA-velocity estimates seems suboptimal (particularly given their biological importance). In fact, one wonders whether simple strategies (e.g. dividing the time series into chunks) would not work.

- Personally, I think RNA velocity, while appealing as a concept, is a bit over-hyped, and there are many aspects I do not understand. So I welcome the effort of the paper to somewhat delineate better what might cause failure. But one aspect that has me permanently baffled is the use of RNA-velocity on 10x polyA trap data sets (which is the case here). If my understanding is correct, most of the signal should come from the terminal regions of transcripts, and therefore one would not expect to see so many intronic reads in the first instance. This seems to me a much stronger model violation than changes in kinetics. I wonder whether the authors should not take the opportunity to also extend their discussion to other potentially important issues with the application of RNA velocity to scRNA-seq data sets (another important issue is the assumption of splicing kinetics to be constant and equal across all genes, while there are plenty of experiments demonstrating great variability in splicing kinetics).

**Authors Response**

**Point-by-point responses to the reviewers’ comments:**

We would like to thank the reviewers for all the constructive comments, see below our point-by-point response (responses are enclosed and highlighted with ***). Complementary figures with additional analyses and a similar text were included in the Personal Cover file also uploaded in the present revised submission.

_____________________________________________________

Reviewer #1:

In this manuscript, Barile et al. applied RNA velocity (an approach based on the ratio between unspliced and spliced RNA) to their previously established temporal scRNAseq data of mouse gastrulation and organogenesis with the goal of predicting the directionality of differentiation. Interestingly, they found that while scVelo was able to correctly predict some differentiation trajectories, it also failed on others, particularly erythropoiesis where it predicts a backward differentiation from later to earlier cell populations. Furthermore, the authors obtained the same result on another scRNAseq dataset from human fetal liver erythropoiesis. Then, they went on to demonstrate that this failure in predicting the correct direction of differentiation was due to specific genes (the MURK genes) that abruptly increase their transcription rate mid-way through erythroid differentiation, violating the assumption of a constant transcriptional rate that underlies sc Velo. Importantly, the authors identified those genes and showed that removing them is sufficient to correct the trajectory direction as inferred by scVelo. Finally, using their chimera-seq approach with Gata1 KO they demonstrate that Gata1 is involved in regulating some of the MURK genes. Furthermore, they found that the Gata1 KO leads to a block of erythroid differentiation and an increase in megakaryocytic differentiation. Finally, they detected an upregulation of Pu1 consistent with the Gata1/Pu1 antagonism.

Overall, this is an elegant and important study with several new findings. The finding of the importance of constant transcription rate for scVelo to correctly predict the direction of erythroid differentiation is very important for anyone planning to use scVelo. The identification of a coordinated change in transcriptional rate for specific genes in late erythroid differentiation is another important finding, permitted by the use of chimera-seq. I only have one question and one comment.

*We were delighted to read the positive feedback by reviewer #1*

Point 1: Beside the "backward" erythroid trajectory, the authors have noticed other, time-related discrepancies in scVelo prediction, including mesoderm and endoderm cells. Are these other discrepancies also due to genes that display an abrupt change in transcription rate?

Conversely, I am wondering if the trajectories where scVelo is correct are completely devoid of genes that change their transcription rate? In other words: can velocity tolerate a certain number/proportion of genes changing their transcription rate and still be correct? These are important questions when deciding whether scVelo can be used in specific cases, and/or whether corrective measures could be used to improve scVelo algorithm.

*We agree with the reviewer that this is an interesting and important question. Of note, since we placed this paper as a preprint on Biorxiv at the time of submission, we have already been contacted by other researchers who now recognize that erroneous RNA velocity predictions in their data are due to the exact same phenomenon that we are describing for the first time in our paper. This means that (i) we are not dealing with an isolated observation, and (ii) our paper is likely to have broad impact and attract citations. These anecdotal interactions with other researchers cannot easily be incorporated into a revision. To further explore the points raised by the reviewer, we have therefore performed more analysis of our embryo dataset, exactly along the lines of what the reviewer suggested.*

*We first took a closer look at the trajectory leading to allantois (see new Supplementary Figure 5 in the revised manuscript). The allantois lineage resembles the erythroid trajectory, as it forms a clear trajectory “branching” out of the main developmental landscape (Supplementary Figure 5A and B). However, its “performance” when analysed with the scVelo pipeline was different to the erythroid trajectory. As shown in Supplementary Figure 5A and B, the arrows are pointing backwards (and therefore disagree with real time progression) when the scVelo pipeline is applied to the whole gastrulation dataset/landscape. However, when we rerun scVelo with only the subset of cells belonging to the allantois trajectory, the arrows ended up agreeing with real time progression (Supplementary Figure 5C). Of note, scVelo calculates the set of highly variable genes each time it is initiated. Consequently, a different set of genes goes into the calculation when using the whole dataset as opposed to only the allantois cells. This analysis therefore highlights a couple of important issues:*

*1) scVelo is highly sensitive to what cells/genes are used for its calculation, which will be different when the landscape is considered as a whole vs when a specific trajectory is isolated.*

*2) This might be taken as evidence that there isn’t a big problem, because as long as you choose the “right” cells, predictions are good. However, we would argue that the opposite is true, because many researchers want to use the Velocity framework to discover trajectories, which means that they would not know beforehand which cells to choose.*

*Figure r1. Identification of MURK genes in two additional developmental trajectories. A. Pijuan-Sala et al. (2019) layout with allantois and NMP cell types highlighted. B. Close-up of regions highlighted in (A). Left plots: cells colored by cell-type; right plots: cells colored by collection time-point. White arrows: scVelo predictions running “backwards” in time. C. UMAP representation of allantois cell subset. The overlaying arrows result from applying scVelo to this cell subset alone. D. Phase plots of two representative allantois MURK genes, with scVelo model prediction overlaid. Cells are colored by collection time-point. E. UMAP representation of cells belonging to the NMP trajectory, colored by collection time-point. The overlaying arrows result from applying scVelo to this cell subset, with “backwards” predictions at later time-points. F. Phase plots of two representative NMP MURK genes, with scVelo model prediction overlaid. G. Same UMAP as in E, overlaying arrows result from applying scVelo to this cell subset after NMP MURK gene exclusion. H. Fitting parameter change models to Krt8 (allantois MURK gene) and to Hoxa7 (NMP MURK gene). Grey line: model of constant parameters over latent time (scVelo assumption); red line: model of transcription rate change over latent time. See also Supplementary Note. I. Distribution of scVelo likelihood scores of highly variable genes for each trajectory subset (allantois, NMPs, erythroid), and MURK genes alone. kde: kernel density estimation. p-values were calculated using two-sided Kolmogorov-Smirnov test, showing that the distribution of MURK genes is significantly different from the remaining HVGs in the case of the erythroid trajectory, in contrast to the other two trajectories considered, where likelihood distributions are similar.*

*We refer to this new analysis in a new section within the discussion, where we now provide a broader discussion of issues to be taken into account when using the Velocity framework.*

*Despite of the clear worries about the “stability” of scVelo predictions, we nevertheless proceeded with analysis of the allantois trajectory to address the other specific question of the reviewer (e.g. if scVelo predicts a trajectory in agreement with real-time sampling, are there still genes showing changes in expression rate?). For this we applied our strategy to identify MURK genes and retrieved 121 allantois MURK genes, including Krt8, essential for the development of extra-embryonic mesodermal tissues (Supplementary Figure 5D). While this result answered the question by the reviewer, we thought that analysis of an additional trajectory was warranted at this point.*

*We therefore applied the same analyses to the trajectory leading from epiblast to neuromesodermal progenitors (NMPs, see Supplementary Figure 5A and B), a trajectory that we previously calculated using Waddington Optimal Transport (see Guibentif et al. Dev Cell. 2021). In this case, the scVelo prediction was still running backwards at the end of the trajectory even when using only the cells belonging to the trajectory (Supplementary Figure 5E). By running our MURK identification pipeline, we retrieved 126 NMP MURK genes. Similarly to what we had observed in the erythroid trajectory, several of the retrieved genes have previously been shown to play a role in NMP development and regulation, namely homeobox transcription factors Hoxa7 and Hoxb9 (Supplementary Figure 5F). As for the erythroid lineage, re-running scVelo upon removal of the identified NMP MURK genes improved the directionality of the arrows (Supplementary Figure 5G).*

*Taken together therefore, this analysis shows:*

*1) MURK genes are not an isolated phenomenon, but can be found across multiple distinct trajectories*

*2) scVelo predictions are highly sensitive to which subset of cells/genes are used for the analysis*

*3) Removal of MURK genes can improve predictions, and is therefore advisable when the true direction of differentiation is not known*

*4) Overall, a great degree of caution needs to be used with the interpretation of scVelo output.*

*These points have all been incorporated into the new discussion paragraph in our revised manuscript.*

*The reviewer also asked whether MURK genes in other contexts were driven by changes in transcription rate, or could it be splicing or RNA degradation rates. We believe that all three parameters may be found to be changing dynamically if we were to examine a broad range of systems. However, in the MURK genes identified in the allantois and NMP trajectories, our modelling approach demonstrated that incorporating dynamic changes in transcription rate was the key parameter change needed to improve the model fit to the experimental data, with a clear improvement compared to the scVelo assumption of constant parameters (see Supplementary Figure 5H).*

*Having performed the above analysis, we realized that it raised an obvious follow-on question to the reviewer’s points, namely why is the effect of the MURK genes bigger in the erythroid trajectory than the other two analysed here? To answer this point, we investigated the distribution of the likelihood score given to each gene by scVelo; this is a score that reflects how well the spliced/unspliced counts for a given gene match with the underlying model assumptions. This analysis showed that although there are indeed MURK genes in the allantois and NMP trajectories showing changes in expression rate, their likelihood score distributions were in the same range as the non-MURK genes. By contrast, a substantial proportion of the MURK genes identified in the erythroid trajectory had higher likelihood values than the erythroid non-MURK genes (Supplementary Figure 5I), which explains the drastic effect of MURK genes in the Erythroid trajectory. For completeness, we are including this analysis also in the new supplementary figure.*

*Taken together, these analyses using additional trajectories demonstrate that*

*1. scVelo is sensitive to the input of highly variable genes, and interpretation of the results will be facilitated in the cases where cells belonging to a given developmental trajectory can be extracted and analyzed separately (which however is actually rather counterintuitive, because it means using scVelo is safest in situations where we already know the trajectory anyway!).*

*2. In the instances where scVelo predictions agree with real-time sampling and biology, there still may be genes displaying changes in transcription rates.*

*3. According to our analyses in three different trajectories, the tolerance of scVelo to the existence of MURK genes depends on their fit to the transcription /splicing /degradation model that underlies scVelo (likelihood) - MURK genes with low likelihood levels will have less impact on the scVelo predictions and will be less likely to lead to erroneous predictions.*

*We have now included the following section in the Discussion:*

*Highlighted in lines 388-402:*

*“To verify whether other developmental processes beyond erythropoiesis may involve time-dependent changes of gene expression parameters, we interrogated two additional trajectories where application of scVelo to the whole Atlas reference had resulted in arrow predictions contrary to real-time progression (see Supplementary Figure 5). These analyses led us to conclude that 1. MURK genes are not restricted to erythropoiesis but can be found across multiple trajectories, including where scVelo predictions are correct; 2. scVelo predictions are highly sensitive to which subset of cells/genes are used for the analysis, which will be different when the landscape is considered as a whole vs when a specific trajectory is isolated; 3. Removal of MURK genes can improve predictions, and is therefore advisable when the true direction differentiation is not known; 4. Overall, a great degree of caution needs to be used with the interpretation of scVelo output; 5. Going forward, rather than simply eliminating MURK genes, future incarnations of the Velocity framework should accommodate the possibility of dynamic changes in parameters, whereby the identification of dynamically changing rates of gene expression may illuminate previously unrecognized aspects of the underlying biological processes.”*

*We furthermore included a detailed legend to the new Supplementary Figure 5:*

*Highlighted in lines 840-875:*

*“Identification of MURK genes in two additional developmental trajectories. A. Pijuan-Sala et al. (2019) layout with allantois and NMP cell types highlighted, where, as for the erythroid trajectory analysed in Figures 1-3, scVelo arrows run contrary to real-time sampling. See Figure 1 for full legend. B. Close-up of regions highlighted in (A). Left: cells colored by cell-type; right: cells colored by collection time-point. White arrows: scVelo predictions running “backwards” in time. C. UMAP representation of allantois cell subset. The overlaying arrows result from applying scVelo to this cell subset alone. In contrast to the predictions resulting from applying scVelo to the whole dataset (see A and B), implementing scVelo (including highly variable genes calculation) to this cell subset alone resulted in correct predictions, with scVelo arrows now in agreement with real-time sampling. This shows that scVelo is highly sensitive to the cell subset, and associated highly variable genes, used in the calculations. D. Phase plots of two representative allantois MURK genes, with scVelo model prediction overlaid. Cells are colored by collection time-point. E. UMAP representation of cells belonging to the NMP trajectory, previously calculated in (31), colored by collection time-point. The overlaying arrows result from applying scVelo to this cell subset, with “backwards” predictions at later time-points. F. Phase plots of two representative NMP MURK genes, with scVelo model prediction overlaid. G. Same UMAP as in E, overlaying arrows result from applying scVelo to this cell subset after NMP MURK gene exclusion, which improved the directionality of the arrows. H. Fitting parameter change models to Krt8 (allantois MURK gene) and to Hoxa7 (NMP MURK gene). Grey line: model with constant parameters over latent time (scVelo assumption); red line: model with transcription rate change over latent time. This analysis suggests that in NMP and allantois, MURK genes may also be driven by transcription rate changes. See also Supplementary Note. I. Distribution of scVelo likelihood scores of highly variable genes for each trajectory subset (allantois, NMPs, erythroid), and MURK genes alone. The likelihood score reflects how well the spliced/unspliced counts for a given gene match with the underlying model assumptions. While the two curves are similar in the case of the allantois and NMP trajectories, a substantial proportion of the MURK genes identified in the erythroid trajectory had higher likelihood values than the remaining erythroid HVGs. p-values were calculated using two-sided Kolmogorov-Smirnov test, showing that the distribution of MURK genes is significantly different from the remaining HVGs in the case of the erythroid trajectory, in contrast to the other two trajectories considered, where likelihood distributions are similar. The high likelihood scores of erythroid MURK genes may explain their pronounced effect on arrow directionality upon scVelo implementation. kde: kernel density estimation.”*

Point 2: In the discussion, it is suggested that Gata1 is important for both cell fate choice (because of the increased in Pu1 expression and megakaryocyte differentiation) and cell fate stabilization (because of the gata1-dependent expression of some effector genes in late erythroid cells). While I agree that the data strongly support a cell fate decision role for Gata1, there is no evidence that the increased expression of effector genes (e.g. the globin genes) in late erythroid cells plays any role in cell fate stabilization. I would suggest to remove this from the Discussion.

*We agree with the reviewer; our use of terminology here was not precise enough, because as pointed out by the reviewer, expression of effector genes is downstream of cell fate decision making. We have therefore removed this part of the discussion as suggested by the reviewer.*

Minor point: On Figures 1 and 2, the arrows from scVelo are very difficult to see. It would help to increase their size.

*We agree with the reviewer that the arrows are difficult to see. While line thickness can be increased, the size of the arrowheads cannot be changed in the software. We thus opted to improve the Figure resolution, and believe this improved figure readability.*

Reviewer #2: The paper by Barile et al presents an interesting and innovative interrogation of a large scRNA-seq data set of mammal erythropoiesis. The most innovative part of the study, in my opinion, is the use of RNA velocity in a "prospective" fashion, i.e. to predict cell transitions and, crucially, to exploit predictive failures to gain new insights in the biology. The authors observe a systematic failure of scVelo to correctly predict some transitions, and then attempt to reconstruct the source of this discrepancy by identifying genes whose expression behaviour appears to violate the modelling assumptions (constant expression kinetics) underlying the model. In doing so, they identify 89 Multiple Rate Kinetics (MURK) genes which exhibit a sharp change in kinetics. Removing these genes greatly improves the performance of scVelo, and also enables the authors to derive several interesting biological observations.

Overall, the paper is well written and interesting, and imo deserves publication. There are two major contributions: the identification of the cluster of MURK genes might be of biological value in itself (I am not an expert on this and I will leave it to others to comment on the more bio aspects of the paper). But to me the more interesting aspect is the use of model failure (scVelo) to learn something new. On this I have a few comments:

- the derivation of the "method" to identify the MURK genes seems mostly heuristic. Which is fine, but I would then want to understand how sensitive it is to arbitrary parameters (e.g. what if, instead of requiring the slope to be positive, one would require it to be greater than say 0.1? what if the p-value threshold is taken to be 0.01 instead of 0.05, or 0.1?).

*We agree with the reviewer that the sensitivity of the method to parameters should be assessed. We have therefore now repeated the analysis using three additional settings, see Figure r2, included in the personal cover document. The values used in the manuscript are in the top grey cells.*

*Figure r2. MURK gene identification in erythroid development, with distinct p-value and slope thresholds. The table on the left shows the number of MURK genes retrieved with each of the selection criteria. On the right are the GO-term enrichment analysis results, comparing panel in Figure 3D with the same analysis using the MURK genes retrieved with a slope threshold s>0.1.*

*Of note, the absolute value of the slopes are not comparable among genes, because it depends on the magnitude of spliced/unspliced counts. In order to use 0.1 as a threshold following the reviewer’s suggestion, we thus scaled the spliced and unspliced counts between 0 and 1, these results are in bold.*

*Changes in p-value threshold had a marginal impact on the number of MURKs retrieved (by ~10%), however setting a threshold in the slope decreased the number of MURKs by 42% (from 89 to 52 MURK genes).*

*To verify whether this would have changed the outcome of our paper in terms of erythroid biology, we re-ran GO-term enrichment analysis on this more stringent MURK gene set. We observed that the GO-terms are very similar to our original results, with MURK genes retrieved using these new parameters also reflecting molecular processes both essential and specific to red blood cell maturation.*

- Ultimately, I imagine one could conceive a more principled way to correct deviations from model assumptions; throwing away the MURK genes in RNA-velocity estimates seems suboptimal (particularly given their biological importance). In fact, one wonders whether simple strategies (e.g. dividing the time series into chunks) would not work.

*We thank the reviewer for bringing this up. We would like to note that our current strategy to model MURK genes already discretizes the time series; however the current scVelo implementation does not support this method to calculate trajectories. We agree with the reviewer that a further optimization of the model to account for deviations from the current assumptions is required, and its implementation needs to allow for these adjustments. In particular, for each gene, scVelo should implement time-dependent rates for every parameter. However, redesigning the scVelo tool would go beyond the scope of the present paper. We have now included these considerations in the Discussion section:*

*Highlighted in lines 399-402:*

*“Going forward, rather than simply eliminating MURK genes, future incarnations of the Velocity framework should accommodate the possibility of dynamic changes in parameters, whereby the identification of dynamically changing rates of gene expression may illuminate previously unrecognized aspects of the underlying biological processes.”*

- Personally, I think RNA velocity, while appealing as a concept, is a bit over-hyped, and there are many aspects I do not understand. So I welcome the effort of the paper to somewhat delineate better what might cause failure. But one aspect that has me permanently baffled is the use of RNA-velocity on 10x polyA trap data sets (which is the case here). If my understanding is correct, most of the signal should come from the terminal regions of transcripts, and therefore one would not expect to see so many intronic reads in the first instance. This seems to me a much stronger model violation than changes in kinetics. I wonder whether the authors should not take the opportunity to also extend their discussion to other potentially important issues with the application of RNA velocity to scRNA-seq data sets (another important issue is the assumption of splicing kinetics to be constant and equal across all genes, while there are plenty of experiments demonstrating great variability in splicing kinetics).

*We very much welcome the reviewer’s suggestion to provide some more discussion of the RNA velocity concept in our paper, and have therefore inserted a few sentences into the revised discussion.*

*This includes:*

*i) some speculation where the unspliced reads actually come from, given that the RNA-Seq platform used here and by many others (10X; 3’) should capture mRNA near the polyA site. The most likely explanation in our opinion is priming of the oligo dT on small stretches of A’s present in the introns. We have seen relatively more intronic reads in Smartseq2 single cell RNA-Seq, which makes sense as tagmentation of the entire reverse-transcribed cDNA is used to make fragments for Illumina sequencing.*

*ii) Refer back to the results presented in our paper here, where we show that the use of MOFA (multiomics Factor Analysis) demonstrates that unspliced reads do have the ability to make a meaningful contribution to the interpretation of complex single cell RNA-Seq datasets*

*iii) A brief discussion of variability in splicing kinetics, how this is known to play an important role both in normal and pathological contexts, and needs to be taken into account as another potential confounder when applying the RNA velocity framework.*

*Highlighted in lines 403-413*

*“One of the major attractions of current usage of the RNA velocity framework is that the added information on unspliced reads comes essentially “for free”, as it is extracted from the raw scRNA-Seq counts. It is however worth remembering that technologies reliant on oligo dT priming are not designed to capture intronic reads with high efficiency, a problem exacerbated when using current droplet methods that sequence specifically either the 3’ or 5’ ends of genes, but not the rest. A likely reason for the capture of intronic reads may be the priming of oligo dT onto small stretches of A present in introns, but there certainly seems scope for the development of future methods specifically designed to increase the capture of unspliced / intronic sequences. It is noteworthy however that our MOFA analysis in Figure 2 fully supports the notion that even with current datasets, the unspliced reads alone provide a degree of lineage discrimination, and contribute to a substantial proportion of the variance when used together with the spliced reads.”*

*Highlighted in lines 417-420*

*“Furthermore, splicing heterogeneity and variable splicing kinetics, known to play an important role both in normal and pathological contexts (reviewed in 49), need to be taken into account as potential confounders when applying the RNA velocity framework.”*

**Second round of review**

**Reviewer 1**

The authors have addressed all my previous comments and questions.

**Reviewer 2**

I thank the authors for their thoughtful response. I am now satisfied with the paper
